# Predictors of work inability after acute myocardial infarction in Switzerland

**DOI:** 10.1038/s41598-024-63988-8

**Published:** 2024-06-11

**Authors:** Fabio Barresi, Fabienne Foster-Witassek, Hans Rickli, Giovanni Pedrazzini, Marco Roffi, Milo Puhan, Holger Dressel, Dragana Radovanovic

**Affiliations:** 1 Division of Occupational and Environmental Medicine, Epidemiology, Biostatistics and Prevention Institute, University of Zurich, Zurich, Switzerland; 2 AMIS Plus Data Center, Epidemiology, Biostatistics and Prevention Institute, University of Zurich, Hirschengraben 84, 8001 Zurich, Switzerland; 3 Department of Cardiology, Cantonal Hospital St. Gallen, St. Gallen, Switzerland; 4 Department of Cardiology, Cardiocentro Ticino, Lugano, Switzerland; 5 Department of Cardiology, Geneva University Hospitals, Geneva, Switzerland; 6 Epidemiology, Biostatistics and Prevention Institute, University of Zurich, Zurich, Switzerland; 7 University Hospital Zurich, Zurich, Switzerland

**Keywords:** Acute myocardial infarction, Return to work, Work inability, Cardiology, Acute coronary syndromes

## Abstract

This study aimed to examine whether acute myocardial infarction (AMI) patients in Switzerland return to work and identify factors associated therewith. Data of 4315 working-age AMI patients enrolled in the Swiss AMIS Plus registry between 01/2006 and 09/2021 with 1-year follow-up and self-reported work status were analyzed. Patient characteristics were compared between those who did not reduce their work hours, those who reduced, and those who were no longer working 1 year after AMI. Multinomial logistic regression was used to analyze independent predictors of working ability. Of the patients, 3204 (74.3%) did not reduce their work hours, 592 (13.7%) reduced and 519 (12.0%) were no longer working 1 year after AMI. Women were more likely to reduce or stop working. Patients who did not reduce were more frequently young and male. Multinomial logistic regression showed that work reduction was associated with female sex and a Killip class > 2 at admission whereas stopping work was associated with female sex and comorbidities. A high rate of AMI patients in Switzerland (88%) return to work 1 year after AMI. Approximately 1 in 8 did not return to work and approximately 1 in 7 reduced their work hours. Important factors associated with reducing or no longer working after AMI were female sex, older age and a higher proportion of comorbidities.

## Introduction

Ischemic heart disease is globally one of the most important causes of death^1^. The WHO describes ischemic heart disease as “The world’s biggest killer”^2^. It is responsible for 16% of the world’s total deaths and in the year 2019 caused 8.9 million deaths worldwide^2^.

In Switzerland, cardiovascular diseases are responsible for over 20,000 deaths every year, which accounts for around a third of all deaths nationwide^3^. In 2019, approximately 17.5% of total deaths in Switzerland were caused by ischemic heart disease^1^.

It was estimated that approximately 45% of patients affected by acute myocardial infarction (AMI) are of working age^4^. Return to work and being able to remain employed after AMI are important markers of functional status, and are associated with individual self-esteem and societal costs^5,6^. The European Cardiovascular Disease Statistics of 2017 stated that in 2015, production losses due to mortality and morbidity associated with cardiovascular disease cost the European Union 54 billion Euro with 42% of this due to illness in those of working age^7^.

In Switzerland, there is lack of knowledge concerning the impact of acute myocardial infarction (AMI) on the ability to continue working. Our aim was therefore to describe the ability to return to work (RTW) after AMI in Switzerland as well as to identify factors associated therewith using data of the nationwide registry AMIS Plus (**A**cute **M**yocardial **I**nfarction in **S**witzerland).

## Methods

AMIS Plus is a large, prospective national registry in Switzerland that has been collecting hospital data on the whole spectrum of patients with acute coronary syndrome (ACS), including AMI and unstable angina. It was founded in 1997 by the Swiss Societies of Cardiology, Internal Medicine and Intensive Care^8^. Since 1997, 84 of 104 hospitals of all sizes have continuously or temporarily collected data for AMIS Plus using a standardized questionnaire filled out by the treating physician or a trained study nurse in the treating hospital. Since 2006, patients were additionally contacted by phone 1 year after the ACS for a follow-up if they gave their informed consent. This study is in accordance with the Declaration of Helsinki regarding investigations on humans and was approved by the Swiss National Ethical Committee for Clinical Studies, the Board for Data Security and all cantonal ethic committees approved the registry (NCT 01,305,785).

Data on self-reported work status before and 1 year after AMI of working-age AMI patients who were contacted by the AMIS Plus registry between 01/2006 and 09/2021 for the follow-up 1 year after hospital admission were analyzed. In Switzerland, the retirement age is 64 years for women and 65 years for men, therefore only data of women < 64 and men < 65 years of age at the time-point of follow-up were selected. Patients not working before and after AMI were excluded from data analysis.

Follow-up was carried out 1 year after discharge by telephone interview by trained AMIS Plus data center staff. Patients were asked about their employment status before and 1 year after the AMI and in case of employment, they were asked the percentage they worked at the respective time points. In Switzerland, a full-time (100%) workload refers to 42 h per week. For this study, work was defined as paid work excluding, for example, informal caregiving or housework.

According to the self-reported work hours before and 1 year after AMI, the patients were divided into 3 groups: patients who did not reduce their work hours; those who reduced their work hours; and patients no longer working 1-year after AMI. Additionally, patients were asked during the follow-up interview about rehospitalizations, complications, rehabilitation participation, newly diagnosed illnesses, complications due to medications and also about their subjective health status compared to the time before AMI.

Information on the clinical status at admission, diagnosis, comorbidities and risk factors was obtained from the main questionnaire filled out by the treating hospitals after the index event according to the following definitions: AMI was defined according to The Fourth Universal Definition of Myocardial Infarction^9^ and patients were further categorized as having ST-segment elevation MI (STEMI) if the initial ECG showed an ST-segment elevation and/or new left bundle branch block. Non-STEMI (NSTEMI) included patients with ST-segment depression or T-wave abnormalities in the absence of ST-elevation on the initial ECG. Killip classification was used to describe the clinical status at admission ranging from a Killip class 1 describing patients with no clinical signs of heart failure to a Killip class 4 describing patients with cardiogenic shock^10^. Dyslipidemia, arterial hypertension and diabetes were considered if the patient was previously treated for such a condition and/or diagnosed by a physician. Patients were defined as smokers if the patients were smokers at the time of the cardiovascular event. Comorbidities were defined according to the Charlson Comorbidity Index (CCI)^11,12^.

### Statistical analysis

Using the Kruskal–Wallis rank sum test, the Chi-square test or the Fisher's exact test we compared patient characteristics between the three groups. Multinomial logistic regression with the reference group “no reduction” was used to analyze the independent impact of comorbidities and cardiac function on work stop or work reduction, respectively. All comorbidities or cardiac function variables with a significant group difference in the descriptive statistics were included in the model. Temporal trends of employment changes were depicted using smoothed line plots with mean estimates and 95% confidence intervals (CIs). Statistical analyses were performed with R version 4.0.5 (The R Foundation for Statistical Computing, 2021) and a *P*-value < 0.05 was considered statistically significant.

## Results

**Figure 1:**
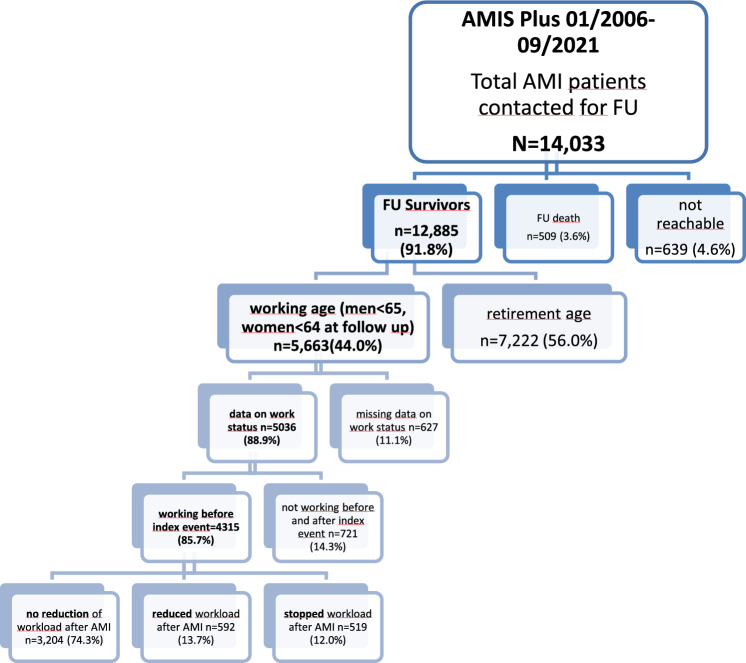
Overview of AMI patients with regard of working status (FU=Follow up).

For the period 2006 to 2021, 14,033 AMI patients had consented to the 1-year follow up, and of these, 12,885 were reachable (Fig. 1). Among these 12,885 AMI patients, 5663 (44.0%) were of working age. For 627 patients, information on working status was incomplete and 721 patients did not work before and after AMI, therefore 4315 AMI patients could be included in this analysis. The follow-up was performed after a median (IQR) time of 400 (375; 441) days. The median (IQR) age was 54.4y (49.3y, 58.6y) and 3772 (87.4%) were males. The median (IQR) age of men was 54.4y (49.2y, 58.7y) and 54.4y (50.1y, 58.1y) for women.

**Figure 2:**
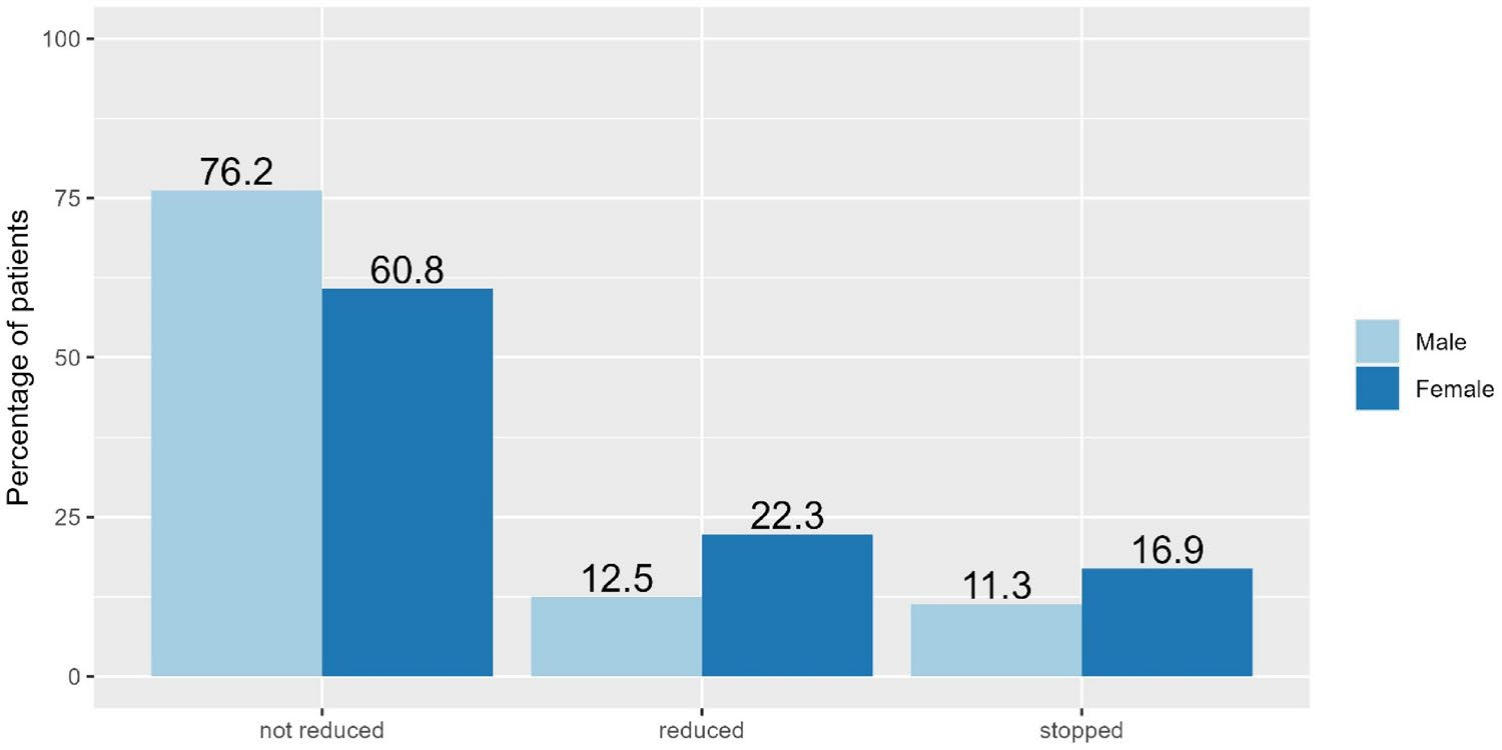
Distribution of men and women in the three groups (in percent with respect to the total of men and women respectively).

The 1-year follow-up showed that 3204 (74.3%) patients did not reduce their work hours, 592 (13.7%) reduced and 519 (12.0%) stopped working (Fig. 1). Patients who did not reduce their work hours, were younger (median age (IQR): 53.8y (48.7y, 58.0y)) than those who reduced and who stopped work (56.5y (51.0y, 60.3y) and 56.5y (51.3y, 60.6y) respectively), and *p* < 0.001 over all groups (Table 1). There was a statistically significant association between sex and the three groups, *p* < 0.001 (Table 1). Men were more likely to not reduce work, and women were more likely to reduce or stop working after 1 year (Table 1). Figure 2 shows the distribution of men and women in the three groups.

**Table 1:** Baseline characteristics of AMI patients according to working status 1 year after acute myocardial infarction.

Characteristics	N	Overall, N = 4,315^1^	not reduced, N = 3,204^1^	reduced, N = 592^1^	stopped, N = 519^1^	*p*-value^2^
Age, years	4,315	54.4 (49.3, 58.6)	53.8 (48.7, 58.0)	56.5 (51.0, 60.3)	56.5 (51.3, 60.6)	** < 0.001**
Sex	4,315					** < 0.001**
Male		3,772 (87.4%)	2,874 (89.7%)	471 (79.6%)	427 (82.3%)	
Female		543 (12.6%)	330 (10.3%)	121 (20.4%)	92 (17.7%)	
Insurance coverage	4,276					**0.002**
Basic		3,503 (81.9%)	2,564 (80.7%)	496 (84.5%)	443 (86.4%)	
Semiprivate/private		773 (18.1%)	612 (19.3%)	91 (15.5%)	70 (13.6%)	
BMI	4,070	26.8 (24.5, 29.7)	26.8 (24.5, 29.6)	26.6 (24.4, 29.7)	27.3 (24.6, 30.1)	0.2
Pre-hospital resuscitation	4,315	193 (4.5%)	131 (4.1%)	41 (6.9%)	21 (4.0%)	**0.008**
Killip class	4,307					
Class I		4,069 (94.5%)	3,054 (95.5%)	538 (91.0%)	477 (92.1%)	
Class II		131 (3.0%)	85 (2.7%)	22 (3.7%)	24 (4.6%)	
Class III		21 (0.5%)	13 (0.4%)	7 (1.2%)	1 (0.2%)	
ClassIV		86 (2.0%)	46 (1.4%)	24 (4.1%)	16 (3.1%)	
Killip class > 2	4,307	107.0 (2.5%)	59 (1.8%)	31 (5.2%)	17 (3.3%)	** < 0.001**
Diagnosis	4,315					0.6
STEMI		2,848 (66.0%)	2,102 (65.6%)	398 (67.2%)	348 (67.1%)	
NSTEMI		1,467 (34.0%)	1,102 (34.4%)	194 (32.8%)	171 (32.9%)	
Current smoker	4,123	2,322 (56.3%)	1,718 (56.2%)	297 (53.1%)	307 (60.8%)	**0.040**
Hypertension	4,129	1,922 (46.5%)	1,371 (44.7%)	282 (49.6%)	269 (54.5%)	** < 0.001**
Dyslipidemia	3,887	2,372 (61.0%)	1,753 (60.4%)	320 (61.5%)	299 (64.0%)	0.3
Diabetes mellitus	4,197	471 (11.2%)	318 (10.2%)	73 (12.7%)	80 (16.0%)	** < 0.001**
Obesity (BMI > 30)	4,070	930 (22.9%)	675 (22.4%)	129 (23.1%)	126 (25.6%)	0.3
Past history of AMI	4,269	399 (9.3%)	271 (8.6%)	61 (10.4%)	67 (13.1%)	**0.003**
Cardiac insufficiency	4,267	19 (0.4%)	11 (0.3%)	3 (0.5%)	5 (1.0%)	0.11
Peripheral vascular disease	4,267	48 (1.1%)	28 (0.9%)	10 (1.7%)	10 (1.9%)	**0.038**
Cerebrovascular disease	4,267	45 (1.1%)	26 (0.8%)	7 (1.2%)	12 (2.3%)	**0.007**
Moderate to severe renal disease	4,267	40 (0.9%)	26 (0.8%)	8 (1.4%)	6 (1.2%)	0.3
CCI > 1	4,267	258 (6.0%)	157 (5.0%)	46 (7.8%)	55 (10.7%)	** < 0.001**
Hospital duration	4,315	4.0 (3.0, 6.0)	4.0 (3.0, 6.0)	5.0 (3.0, 7.0)	5.0 (3.0, 7.0)	** < 0.001**

^1^Median (IQR); n (%).

^2^Kruskal-Wallis rank sum test; Pearson's Chi-squared test; Fisher's exact test.

IQR = Interquartile range. CCI = Charlson comorbidity index.

Significant values are in bold.

Patients who reduced work showed worst cardiac function at AMI reflected in the highest rates of Killip class > 2 (no reduction: 1.8%, reduced: 5.2%, stopped: 3.3%, *p* < 0.001) and resuscitation before admission (no reduction: 4.1%, reduced: 6.9%, stopped: 4.0%, *p* = 0.008). Patients who stopped work had the most comorbidities, such as past AMI (no reduction: 8.6%, reduced: 10.4%, stopped: 13.1%, *p* = 0.003), hypertension (no reduction: 44.7%, reduced: 49.6%, stopped: 54.5%, *p* < 0.001), diabetes mellitus (no reduction: 10.2%, reduced: 12.7%, stopped: 16.0%, *p* < 0.001) and cerebrovascular disease (no reduction: 0.8%, reduced: 1.2%, stopped: 2.3%, *p* = 0.007) (Table 1).

Patients indicating unchanged or better subjective health status at the 1-year follow-up after AMI were more likely to not reduce work 1 year after AMI, whereas patients indicating a subjectively worse health status were more likely to stop working (*p* < 0.001) (Table 2). Patients who were rehospitalized or had a reinfarction after the initial hospitalization were more likely to stop working (no reduction: 17.6%, reduced: 24.8%, stopped: 37.8%, *p* < 0.001 and no reduction: 2.0%, reduced: 3.2%, stopped: 7.6%, *p* < 0.001, respectively). Patients with a defibrillator/pacemaker implantation during follow up were more likely to stop working (no reduction: 0.6%, reduced: 2.1%, stopped: 4.3%, *p* < 0.001 and no reduction: 0.3%, reduced: 0.5%, stopped: 1.4%, *p* = 0.005, respectively). Rehabilitation showed no association with RTW (no reduction: 84.2%, reduced: 86.0%, stopped: 83.7%, *p* = 0.7) (Table 2).

**Table 2:** Follow-up data of acute myocardial infarction patients 1 year after discharge.

Characteristics	N	Overall, N = 4,315^1^	not reduced, N = 3,204^1^	reduced, N = 592^1^	stopped, N = 519^1^	*p*-value^2^
Subjective health status assessment	4,276					** < 0.001**
Worse		1,248 (29.2%)	700 (22.1%)	269 (45.6%)	279 (54.2%)	
Same as before AMI		1,967 (46.0%)	1,612 (50.8%)	204 (34.6%)	151 (29.3%)	
Better		1,061 (24.8%)	859 (27.1%)	117 (19.8%)	85 (16.5%)	
Rehospitalization during follow up	4,306	906 (21.0%)	563 (17.6%)	147 (24.8%)	196 (37.8%)	** < 0.001**
Number of rehospitalizations	382					**0.009**
1		311 (81.4%)	201 (84.1%)	53 (82.8%)	57 (72.2%)	
2		51 (13.4%)	32 (13.4%)	5 (7.8%)	14 (17.7%)	
> 2		20 (5.2%)	6 (2.5%)	6 (9.4%)	8 (10.1%)	
Kind of rehospitalization(s)	863					**0.003**
emergency		305 (35.3%)	171 (31.9%)	46 (33.6%)	88 (46.3%)	
planned		520 (60.3%)	345 (64.4%)	83 (60.6%)	92 (48.4%)	
emergency and planned		38 (4.4%)	20 (3.7%)	8 (5.8%)	10 (5.3%)	
Stroke during follow up	4,298	14 (0.3%)	3 (0.1%)	4 (0.7%)	7 (1.4%)	** < 0.001**
Reinfarction during follow up	4,293	122 (2.8%)	64 (2.0%)	19 (3.2%)	39 (7.6%)	** < 0.001**
Defibrillator implantation during follow up	4,280	54 (1.3%)	20 (0.6%)	12 (2.1%)	22 (4.3%)	** < 0.001**
Pacemaker implantation during follow up	4,482	20 (0.5%)	10 (0.3%)	3 (0.5%)	7 (1.4%)	**0.005**
Rehospitalization due to another disease	1,976					** < 0.001**
No		1,702 (86.1%)	1,261 (88.2%)	259 (84.4%)	182 (76.2%)	
Yes		271 (13.7%)	167 (11.7%)	48 (15.6%)	56 (23.4%)	
Planned		3 (0.2%)	2 (0.1%)	0 (0.0%)	1 (0.4%)	
Any new illnesses diagnosed	1,971	195 (9.9%)	115 (8.1%)	36 (11.7%)	44 (18.6%)	** < 0.001**
Rehabilitation during follow up	2,056	1,736 (84.4%)	1,259 (84.2%)	271 (86.0%)	206 (83.7%)	0.7
Complications due to medication	2,336	300 (12.8%)	217 (13.1%)	52 (14.6%)	31 (9.5%)	0.11

^1^n (%).

^2^Pearson's Chi-squared test; Fisher's exact test.

Significant values are in bold.

**Figure 3:**
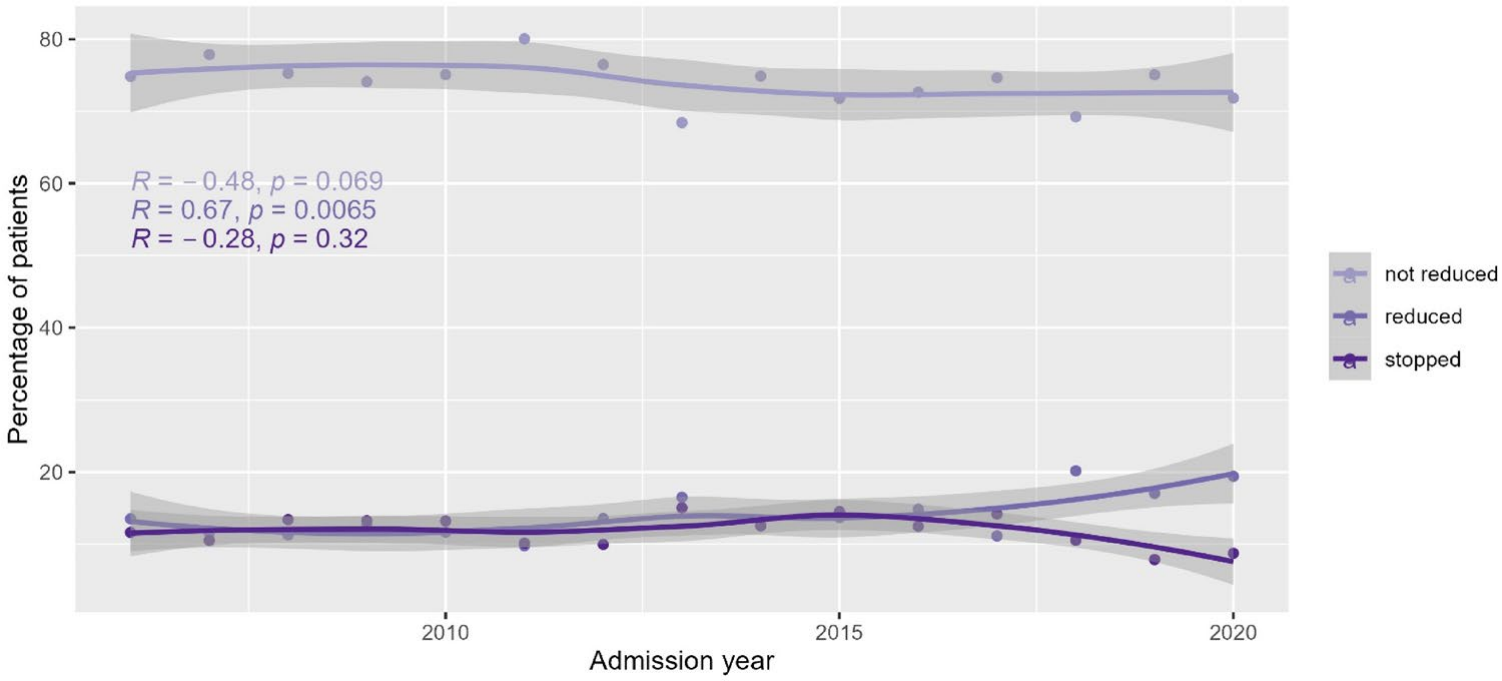
Trends of employment rates after acute myocardial infarction over time (2006–2021).

Over time (2006–2020), the rate of patients not reducing work after AMI remained stable, whereas the rate of patients stopping work tended to decrease and the rate of patients reducing work tended to increase in the years between 2018 and 2020 (Fig. 3).

**Figure 4:**
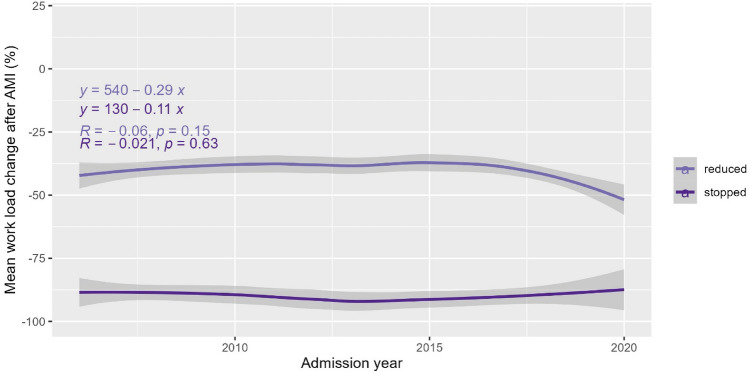
Mean work load change after AMI in patients who reduced work and in patients who stopped work over time (2006–2021).

The mean (SD) work percentages for the no reduction, reduction and work stop groups were 93.5 (19.0)%, 94.1 (15.4)% and 89.8 (21.5)% before AMI and 94.9 (15.7)%, 53.8 (21.9)% and 0 (0)% after AMI. The no reduction group included 92 patients who had increased their work load. The mean change in work percentages in the reduction group (*p* = 0.15) and the work stop group (*p* = 0.63) remained stable over time (2006–2020) (Fig. 4).

Multinomial regressions for the prediction of work reduction and stopping work showed that a work reduction was significantly associated with female sex (OR 2.30; 95% CI 1.80–2.93 *p* < 0.001) and a Killip class > 2 at admission (OR 2.54; 95% CI 1.51–4.27 *p* < 0.001). A work stop was associated with female sex (OR 2.04; 95% CI 1.57–2.67 *p* < 0.001) and comorbidities (past AMI (OR 1.48; 95% CI 1.09–2.01 *p* = 0.011), diabetes (OR 1.61; 95% CI 1.22–2.12 *p* = 0.001), cerebrovascular disease (OR 2.63; 95% CI 1.28–5.42 *p* = 0.009)) (Table 3).

**Table 3:** Results from multinomial logistic regression analysis.

Predictors	Multinomial logistic regression (Reference group: no reduction)			
	Odds ratios	CI	*p*	Response
(Intercept)	0.01	0.00 – 0.02	** < 0.001**	reduced
Age, years	1.05	1.03 – 1.07	** < 0.001**	reduced
Sex [Female]	2.30	1.80 – 2.93	** < 0.001**	reduced
Killip class > 2 [Yes]	2.54	1.51 – 4.27	** < 0.001**	reduced
Resuscitation prior to admission [Yes]	1.37	0.90 – 2.10	0.139	reduced
History of AMI [Yes]	1.20	0.88 – 1.64	0.246	reduced
Diabetes mellitus [Yes]	1.21	0.91 – 1.60	0.201	reduced
Arterial hypertension [Yes]	1.09	0.90 – 1.31	0.400	reduced
Cerebrovascular disease [Yes]	1.32	0.56 – 3.15	0.525	reduced
Peripheral vascular disease (ST III + IV) [Yes]	1.48	0.67 – 3.25	0.334	reduced
(Intercept)	0.01	0.00 – 0.02	** < 0.001**	stopped
Age, years	1.05	1.03 – 1.06	** < 0.001**	stopped
Sex [Female]	2.04	1.57 – 2.67	** < 0.001**	stopped
Killip class > 2 [Yes]	1.65	0.85 – 3.17	0.136	stopped
Resuscitation prior to admission [Yes]	0.88	0.51 – 1.50	0.631	stopped
History of AMI [Yes]	1.48	1.09 – 2.01	**0.011**	stopped
Diabetes mellitus [Yes]	1.61	1.22 – 2.12	**0.001**	stopped
Arterial hypertension [Yes]	1.23	1.00 – 1.50	0.050	stopped
Cerebrovascular disease [Yes]	2.63	1.28 – 5.42	**0.009**	stopped
Peripheral vascular disease (ST III + IV) [Yes]	1.28	0.56 – 2.94	0.560	stopped
Observations	4016			
R^2^ / R^2^ adjusted	0.100 / 0.100			

*CI* = *95%-Confidence interval. p* = *p-value. ST III* + *IV* = *Stage III & Stage IV.*

Significant values are in bold.

## Discussion

The data from the AMIS Plus database in Switzerland showed a RTW rate of 88% 1 year after AMI, which means that approximately 1 in 8 did not return to work 1 year after AMI. Patients with comorbidities were more likely to stop working whereas worse cardiac function was associated with a work reduction. Furthermore, our data showed that women were more likely to reduce or stop working after AMI, whereas men were more likely not to reduce working. Multinomial regression showed that both work reduction and stopping work were significantly associated with female sex. To our knowledge, no similar data was published before for Switzerland.

Several studies from different countries reported that male gender was associated with a greater RTW rate after AMI as described in a systematic review from the year 2021^13^ where various reasons have been suggested to explain this observation. For example, men may feel pressured to resume work after AMI in order to support their family financially, and women might have greater responsibilities for provision of family care and undertake more domestic work^13^.

With respect to RTW after AMI, similar RTW rates as found in our data were reported in studies from other countries. In a study conducted in the USA, Spain and Australia with 1680 participants published in the year 2016, a RTW rate of 86% at 12-month follow-up was described^14^. In a nationwide retrospective cohort study conducted in Denmark with 22,394 participants, published in the year 2017, a RTW rate of 91.1% at 12 months post-AMI was found^5^. A prospective cohort study conducted in Italy with 102 participants published in the year 2018 showed a RTW rate of 92.7% 1 year post-AMI^6^. Warraich et al. used data from a registry to assess the prevalence of adverse change in employment between baseline and 1 year post-AMI in a national US cohort and found that at 1 year post-AMI, 10% reported an adverse change in employment (3% working less and 7% no longer working)^15^.

Other studies, however, described lower RTW rates. A prospective cohort study in the Netherlands, published in 2014, showed a RTW rate of 76.9% at 12 months post-AMI^16^. An observational study in France published in 2010 showed a RTW rate of 76% with a mean return time of 134 days (range 7–990 days)^17^. For Germany, a RTW rate of 74% within 2 years was described^18^. Differences in observed RTW rates among countries may be explained for example by differences in policies for paid sick leave, differences in retirement age or differences in availability of cardiovascular rehabilitation services in different countries^13^.

It should be emphasized that RTW rates among studies may not be comparable due to different inclusion criteria, outcome definition or study design. Also, different publication timepoints may have an impact on the reported RTW rate. For example, it may be possible that advancements in AMI care, e.g. improvement of treatments and reduction of complications, have an influence on RTW rates over time. Our analysis showed that the rate of those not reducing work remained stable over time. On the other hand, the rates of those reducing work and those stopping work were almost identical for the years 2006 until 2018, and afterwards a trend of decreasing rate in those stopping work was recognizable while the rate of those reducing work was increasing.

Our data showed that a higher proportion of comorbidities, such as past AMI, hypertension, diabetes mellitus and cerebrovascular diseases as well as rehospitalization or reinfarction after the initial event was more likely associated with stopping work. Similar findings were described in other studies^5,15,19^.

Further factors inhibiting RTW after AMI described in other publications were for example: depression, lower education, cancer, use of antidepressants or anxiolytics, a history of sickness absence, a physically demanding occupation and being a blue collar worker^13,16,20,21^.

The data from the AMIS Plus database showed that approximately 1 in 7 reduced professional activity 1 year after AMI. Work reduction was significantly related to worse cardiac function. Warraich et al. described 3% of patients working less at 1 year post-AMI^15^. This is in stark contrast to our finding of approximately 14% of individuals reducing work after AMI. In a qualitative study conducted in Turkey with 12 male AMI patients and published in the year 2016, it was described that “many of the participants continued to work at the same job by working less” after AMI^22^. In a study conducted in Italy with 253 angioplasty or heart surgery patients it was described that work hours decreased in 57% of the patients^23^, which is a clearly higher rate than what we found.

The observations described here may not be comparable due to different patient collectives and different types of analyzed outcomes. In addition, socio-political and cultural differences in different countries may also influence a patient’s decision to reduce work, making comparisons difficult.

Further, the data from the AMIS Plus database showed that 74.3% did not reduce work 1 year after AMI. Patients not reducing work after AMI were younger than those reducing or stopping work. Other studies reported similar findings^16,17,19,24,25^. As described elsewhere^13^, older patients may opt for an early retirement after experiencing AMI, which may partially explain why older age results as a factor inhibiting RTW. However, Warraich et al. found in their study that only 27 in 492 individuals who reported an adverse change in employment 1 year post-AMI reported retirement^15^.

Our data showed that patients reporting a better subjective health status at the 1-year follow-up were more likely to RTW. This is in line with other studies^25,26^. Further factors facilitating RTW after AMI reported in other studies were for example: high educational level, self-employment status, higher job satisfaction, higher salary, clerical type of work and being married^5,6,14,16,19,27^. In our study, patients not reducing work had the highest rate of semiprivate/private insurance coverage which might, similar to these studies, reflect a better socio-economic status compared to patients reducing or stopping work.

In our study, rehabilitation after the initial event showed no association with the RTW rate. Maybe an association was not detectable because only data of patients who were contacted for follow-up were used and therefore data of other AMI patients undergoing rehabilitation is not present in this analysis. With respect to RTW, the role of rehabilitation in patients with cardiovascular disease seems to be inconclusive. A systematic review and meta-analysis about RTW in cardiovascular patients after cardiac rehabilitation showed in subgroup analyses that the proportion of RTW was higher in white-collar compared to blue-collar workers and that cardiac rehabilitation in out-patients was more effective compared to cardiac rehabilitation in in-patient and usual care^28^.

Lastly, our data showed that 5663 (44.0%) of 12,885 patients with AMI were of working age. This is in line with a previous study describing a rate of approximately 45%^4^, but is slightly lower when compared to the rate of approximately 50% given by a follow-up study conducted in Iran on 200 patients suffering from first AMI attack^27^. It must be considered that the rate of patients of working age in different studies may not be comparable due to different inclusion criteria of patients across different studies and different definitions of working age across different countries.

### Strengths and limitations

For the first time it was possible to analyze the association between several patient characteristics and RTW after AMI for a large number of AMI patients in Switzerland.

However, it must be noted that the focus of the AMIS Plus registry is not on researching RTW issues. Some important data, for example, educational level, professional activity before and after AMI, time until returning to work after AMI, job satisfaction, symptoms of anxiety/depression or the exact reasons for reducing or stopping work are missing in the AMIS Plus database.

## Conclusion

The analysis of the data in the AMIS Plus registry showed that a high rate of patients (88%) return to work 1 year after AMI. Females were more likely to reduce or stop working after AMI, whereas males were more likely not to reduce work. Younger age was an important factor associated with RTW after AMI. Work reduction was significantly associated with worse cardiac function whereas stopping work was associated with comorbidities. Over the observed time period (2006–2021), the rate of patients not reducing work after AMI remained stable. However, since 2018 a trend can be seen in a decreasing rate of patients stopping work after AMI and at the same time an increasing rate of patients reducing work after AMI. Knowledge of factors facilitating or inhibiting RTW after AMI is important in order to adapt healthcare measures, work conditions and policies with the aim to improve the RTW rate after AMI.

## Data Availability

Individual data used for the construction of the AMIS Plus registry are property of the hospitals participating in the AMIS registry and may only be made available by each hospital’s PI and the AMIS Plus Steering Committee. Due to data protection regulations related to the different hospitals involved in this study, the authors do not have authorization to provide unrestricted data access. However, after approval of the AMIS Plus Steering Committee and subsequent negotiation of an individual AMIS Plus module contract with the AMIS Plus Steering Committee, analysis files may be provided to other researchers. Requests must be submitted to Prof. Dr. Hans Rickli, President of the AMIS Plus Steering Committee (hans.rickli@kssg.ch) and Dr. Dragana Radovanovic (Head of the AMIS Plus Data Center, dragana.radovanovic@uzh.ch).
